# Postpartum depression as the main predictor of impaired mother- infant bonding: A prospective study in a low-risk obstetric and neonatal cohort

**DOI:** 10.1371/journal.pone.0347856

**Published:** 2026-05-29

**Authors:** Marielle Ribeiro Feitosa, Saint Clair Gomes Júnior, Emanuella Carneiro Melo, Antonio Brazil Viana Júnior, Paulo César de Almeida, Fernanda Rebelo

**Affiliations:** 1 National Institute of Women, Children and Adolescents Health Fernandes Figueira (IFF), Oswaldo Cruz Foundation (Fiocuz), Rio de Janeiro, Brazil‌‌; 2 Assis Chateaubriand School Maternity (MEAC), Federal University of Ceará (UFC), Brazilian Hospital Services Company (EBSERH), Fortaleza, CE, Brazil; 3 Walter Cantídio University Hospital (HUWC), Federal University of Ceará (UFC), Brazilian Hospital Services Company (EBSERH), Fortaleza, CE, Brazil; 4 State University of Ceará (UECE), Fortaleza, CE, Brazil; Federal University of Santa Maria: Universidade Federal de Santa Maria, BRAZIL

## Abstract

**Introduction:**

The postpartum period imposes complex demands on women, making it essential to examine the mother-infant bond and its predictors to promote maternal and child health and inform public policy.

**Objectives:**

To identify the primary predictors of the mother-infant bond within the first six months postpartum, considering sociodemographic, obstetric, and neonatal factors, as well as symptoms of postpartum depression (PPD).

**Methods:**

A prospective cohort study was conducted with postpartum women in a northeastern Brazilian capital city, assessed at three and six months postpartum. The affective bond was measured using the Postpartum Bonding Questionnaire (PBQ), and PPD symptoms were evaluated with the Edinburgh Postnatal Depression Scale (EPDS). Associations were analyzed using multiple linear regression models and reported as β coefficients (95% confidence intervals).

**Results:**

PPD was the strongest predictor of impaired mother-infant bonding. At the third month postpartum, PPD was significantly associated with total PBQ scores (β = 1.15, 95% CI: 0.93–1.37), impaired bonding (ABF1: β = 0.59, 95% CI: 0.46–0.72), and anxiety about the infant (ABF3: β = 0.28, 95% CI: 0.23–0.33). These associations remained significant at the sixth month postpartum across the same measures: total PBQ (β = 0.84, 95% CI: 0.61–1.08), ABF1 (β = 0.40, 95% CI: 0.26–0.54), and ABF3 (β = 0.24, 95% CI: 0.19–0.30). In contrast, social support emerged as a protective factor, negatively correlating with total PBQ scores (β = −8.53, 95% CI: −13.03 to −4.04) and impaired bonding (ABF1: β = −3.59, 95% CI: −6.26 to −0.93).

**Conclusion:**

These findings identify PPD as a primary risk factor and social support as a crucial protective factor for mother-infant bonding. The results underscore the importance of routine PPD screening and the development of targeted interventions to enhance social support networks for women during the postpartum period.

## Introduction

The mother-infant bond during the postpartum period is characterized by the mother’s capacity to respond appropriately to her infant’s needs and by her positive perception of the infant’s feedback [1,2]. This bond is a dynamic, relational process essential for child development, as it provides a secure and nurturing environment while enhancing maternal self-efficacy [3–5]. Research indicates that the emotional bond formed within the first months of life serves as a protective factor against stressors in child development and lays the foundation for future interpersonal relationships [2,6]. Conversely, disruptions in this bond can cause substantial harm, compromising the mother-child relationship, increasing vulnerability to abuse and neglect, and adversely affecting psychosocial development [7–10]. Although the mechanisms underlying the development of these bonds are not fully elucidated, they are believed to be influenced by neonatal care practices [9,11].‌‌

Previous studies have demonstrated that the establishment of the emotional bond between mother and infant is a dynamic process influenced by multiple external factors. These include social, economic, and cultural predictors such as low educational attainment [12–14], financial insecurity [15,16], and lack of family support [4]. Additionally, obstetric and neonatal factors significantly shape mother-infant bonding, including high-risk or unplanned pregnancies, prematurity, labor complications, inadequate support for skin-to-skin contact and breastfeeding, and mother-infant separation shortly after delivery [6,17,18].

Other factors also appear to contribute to disruptions in the establishment of the mother-infant bond, with postpartum depression (PPD) being particularly prominent [19,20]. PPD is among the most common mental disorders occurring during pregnancy or the postpartum period [21], affecting approximately one in four individuals in low- and middle-income countries [22]. It typically manifests within the first 4–6 weeks postpartum, reaching peak severity within the first six months, and may persist up to one year after childbirth [22,23].

PPD is regarded as a silent epidemic due to the delayed recognition of symptoms, which may emerge gradually or abruptly [24]. Approximately 85% of postpartum women report experiencing some degree of sadness or depressive mood within the first days following childbirth, along with feelings such as fear, anxiety, and a sense of exclusion related to the infant [25]. Despite frequent complaints, women with PPD often remain undiagnosed, primarily owing to healthcare professionals’ limited knowledge or challenges in accurately identifying the signs and symptoms of PPD [19–21,26].

A literature review spanning 2005–2014, encompassing 203 studies from 42 countries, reported that the prevalence of PPD ranged from 1.9% to 82.1% in developed countries and from 5.2% to 74.0% in developing countries. In these regions, PPD prevalence within the first eight weeks postpartum was approximately 35%, declining to 25% by six months postpartum [27]. In southern Brazil, a 2019 cohort study identified suspected PPD in 31% of participants [28], whereas a 2021 study conducted in the Northeast reported a prevalence of 39.13% [29]. The ‘Nascer no Brasil’ study estimated a PPD prevalence of 26.3% across five macro-regions [30]. Notably, these rates may be elevated due to the COVID-19 pandemic [31], which has increased the risk of PPD development and adversely impacted postpartum women’s mental health [32].

Therefore, understanding the mother-infant bond during the postpartum period and its predictors is crucial, as the quality of this relationship is strongly linked to maternal responsiveness and the socioemotional development of children. Research indicates that this bond is influenced by various factors, including socioeconomic conditions, PPD, stress, social support, breastfeeding quality, and obstetric and neonatal experiences. Consequently, identifying the degree of emotional bonding, along with its associated risk and protective factors, is essential for delivering high-quality care to the mother-infant dyad [32,33]. This knowledge facilitates the design of targeted interventions that promote a secure relationship, enhance maternal physical and mental health, and support optimal child development.

Although evidence exists regarding factors influencing the affective bond, significant knowledge gaps persist. The majority of studies have concentrated on the immediate postpartum period and high-risk populations, with a notable lack of longitudinal research examining, in an integrated manner, the predictors of the affective bond among women at low obstetric and neonatal risk [34]. This gap is especially pertinent in socially vulnerable contexts, such as northeastern Brazil. Accordingly, by longitudinally assessing these predictors at three and six months postpartum, this study aims to generate evidence that can inform preventive interventions, enhance comprehensive maternal and child healthcare, reduce health inequities, and strengthen support networks.‌‌

The selection of this topic is justified by the interest in understanding the mother-infant relationship in the formation of the individual and the potential effects of disrupted affective bonding on maternal and child development. Accordingly, this study aims to investigate the association between PPD and other risk factors with the quality of the mother-infant affective bond during the first six months postpartum, a critical period for both the consolidation of the bond and the persistence of PPD symptoms.

## Methods

### Study design, population and location

This cohort study involved breastfeeding women admitted to the rooming-in unit at Assis Chateaubriand Maternity School (MEAC). Eligible participants were recruited during the first postpartum week and followed up at three and six months postpartum.

### Context

MEAC is a care, teaching, and research unit integrated into the Unified Health System (SUS) and part of the Hospital Complex of the Federal University of Ceará (UFC), affiliated with the Brazilian Hospital Services Company (EBSERH). Recognized as a reference center for maternal and child health care at medium and high maternal and fetal risk levels, MEAC has received the Baby-Friendly Hospital Initiative designation and implemented policies such as the Kangaroo Method, the Alyne Network, and the QualiNEO Strategy. The institution also hosts the House of Pregnant, Infant, and Puerperal Women (CGBP) and the Human Milk Bank (BLH) and is acknowledged by the Brazilian Ministry of Health as a Center for Good Practices in Obstetrics and Neonatology [35].

In 2023, MEAC conducted 4,510 deliveries, recording a total of 4,623 births. It is currently the largest public maternity hospital in Ceará, located in northeastern Brazil [35]. This context presents unique methodological relevance, as it integrates maternal and child care across varying levels of complexity with the systematic implementation of humanization policies, evidence-based obstetric and neonatal practices, professional training programs, and research activities. By primarily serving populations experiencing socioeconomic vulnerability, this institutional setting constitutes a strategic environment for investigating the determinants of the mother-infant bond.

### Eligibility criteria and sample size

The eligibility criteria were established to form an initially homogeneous cohort, facilitating a more precise investigation of the association between PPD development, sociodemographic, obstetric, neonatal, and breastfeeding characteristics, and the mother-infant affective bond.

Eligible participants were breastfeeding women aged ≥18 years, hospitalized in rooming-in wards during the first postpartum week, whose infants were born at term and classified as appropriate for gestational age (AGA) according to World Health Organization (WHO) growth curves [36]. Additionally, participants were required to have established exclusive breastfeeding at recruitment (baseline), defined as the infant receiving only breast milk—either directly from the breast or expressed—without any supplementary food or beverages, except for drops or syrups containing vitamins, oral rehydration salts, mineral supplements, or medications, as per WHO guidelines [37]. This criterion was essential to evaluate whether maintaining exclusive breastfeeding over time influences the affective bond, starting from a standardized baseline.

Postpartum women with severe obstetric or neonatal complications, such as significant hemorrhage, eclampsia, birth asphyxia, prematurity, or low birth weight, were excluded to reduce the potential confounding effects of acute labor stress or acute clinical conditions on the assessment of affective bonding and PPD. Similarly, women with a prior history of mental disorders or those currently using psychiatric medications were excluded to ensure that the evaluation of PPD incidence reflected cases emerging specifically during the postpartum period, uncontaminated by pre-existing psychopathology.

Although these restrictive criteria may limit the generalizability of the findings to the broader postpartum population by introducing selection bias toward women at low obstetric and neonatal risk, this approach is methodologically justified as it minimizes potential confounding biases. The resulting gain in internal validity—namely, an enhanced capacity to analyze the associations of interest with reduced interference from external risk factors—outweighs this limitation and provides robust evidence on the specific dynamics of the postpartum period within an optimal health context.

The sample size was calculated using G*Power software, employing the option to assess the association between mother-infant affective bonding and PPD. A post hoc analysis was performed to determine the statistical power achieved with the collected sample, considering the following parameters: two-tailed Fisher’s exact test; proportion in group 1 (impaired affective bonding with PPD, p₁ = 55.0%); proportion in group 2 (impaired affective bonding without PPD, p₂ = 15%); and a significance level (α) of 0.05. Based on these parameters, the estimated sample included 18 participants in group 1 and 98 in group 2, yielding a statistical power of 92.75%.

Postpartum follow-up assessments were conducted at three (T1) and six (T2) months postpartum, with 125 and 116 lactating women participating, respectively, yielding a dropout rate of 7.2%. These time points were selected based on Bowlby’s attachment theory [2], which delineates critical sequential stages in affective bond development. The third month (T1) marks a pivotal phase when the infant begins differentiating stimuli, establishing the qualitative foundations of the emerging affective bond, while the sixth month (T2) signifies the consolidation phase, during which attachment behaviors become clearly directed toward the primary caregiver. This longitudinal design facilitates observation of the dynamic transition and stabilization of the mother-infant affective bond over time.

### Recruiting the participants

Recruitment occurred during the first postpartum week (on average, 6 hours after delivery), following eligibility verification via medical record review. Eligible postpartum women were invited to participate and, after receiving comprehensive information about the study’s objectives and procedures, provided written informed consent. Subsequently, data on maternal characteristics and birth-related variables were collected. At T1 and T2, participants were contacted by telephone to administer follow-up assessments.

### Data collection instruments

The Postpartum Bonding Questionnaire (PBQ) and the Edinburgh Postpartum Depression Scale (EPDS) were employed to assess mother-infant affective bonding (AB) and PPD symptoms, respectively. Both instruments have been validated and translated into Brazilian Portuguese and are commonly used by healthcare professionals and midwives for the early identification of mother-infant bonding disorders [8]. For clarity, mother-infant affective bonding and PPD symptoms will hereafter be referred to as AB and PPD, respectively.

Data collection was conducted by the study researchers from March 2023 to June 2024. Variables including AB, PPD, social support network, and breastfeeding were assessed at the follow-up time points T1 and T2.

#### Mother-infant bonding – PBQ.

The Postpartum Bonding Questionnaire (PBQ) was employed to assess disturbances in the mother-infant emotional bond during the postpartum period. This multidimensional instrument, developed by Brockington et al. (2001) [8], has been validated in multiple countries, including Brazil in 2018 [38]. The PBQ comprises 25 items rated on a six-point Likert scale ranging from 0 (“never”) to 5 (“always”), with total scores ranging from 0 to 125. Higher scores indicate greater impairment in the mother-infant bond, while lower scores reflect better bonding quality.

The PBQ is evaluated through the total score, referred to as bonding disorder (AB total), and four factors: impaired bonding (ABF1), rejection and pathological anger (ABF2), anxiety about the infant (ABF3), and incipient abuse (ABF4). The instrument comprises both positively and negatively worded items, scored according to standardized guidelines. Cut-off values for identifying probable bonding disturbances are ≥ 26 for AB total, ≥ 12 for ABF1, ≥ 13 for ABF2, ≥ 10 for ABF3, and ≥2 for ABF4 [8,38,39].

In this study, the mother-infant emotional bond (total score and subscales) was analyzed as a dichotomous variable, categorized as compromised or preserved.

#### Postpartum Depression – EPDS.

Symptoms of postpartum depression (PPD) were assessed using the Edinburgh Postnatal Depression Scale (EPDS), a screening instrument originally developed by Cox, Holden, and Sagovsky (1987) in the United Kingdom [40]. The scale was translated and validated for use in Brazil by Santos et al. (2007) [41]. The EPDS comprises 10 items, each with four response options scored from 0 to 3, reflecting the frequency and severity of depressive symptoms experienced by the mother over the previous seven days. Total scores range from 0 to 30, with scores ≥13 indicating the probable presence of depressive symptoms consistent with PPD [41]. For this study, PPD was analyzed as a dichotomous variable, categorized as “yes” (presence of depressive symptoms) or “no” (absence of depressive symptoms).

### Covariates

In addition to postpartum depression (PPD) and mother-infant bonding (AB) data, variables related to sociodemographic characteristics, perinatal factors, and breastfeeding practices were analyzed.

Sociodemographic characteristics included place of residence, categorized as residing in the state capital or in the interior. Maternal race/skin color was self-reported and classified as white, black, or brown. Maternal age was dichotomized as <35 years or ≥35 years. Educational level was grouped based on completion of high school (incomplete or complete). Marital status was categorized as “does not live with partner” or “lives with partner.” Monthly family income, reported in multiples of the Brazilian minimum wage at the time of data collection, was dichotomized as ≤1 minimum wage or >1 minimum wage. The presence of self-reported maternal chronic diseases (e.g., arterial hypertension and diabetes mellitus) was treated as a dichotomous variable (no/yes). Maternity leave status was also analyzed dichotomously (no/yes). The number of children was classified as one or two or more.

Perinatal characteristics included type of delivery, categorized as cesarean or vaginal. Maternal and paternal desire for pregnancy were self-reported and analyzed as dichotomous variables (no/yes). Preconception smoking was defined based on self-reported tobacco use prior to pregnancy (no/yes). The number of prenatal visits was categorized as <6 or ≥6, in accordance with Brazilian Ministry of Health recommendations. Infant sex was classified as male or female. The indicator of good neonatal practices was based on maternal reports of immediate skin-to-skin contact and breastfeeding within the first hour of life, classified as “yes” when both practices occurred and “no” when one or neither was reported.

The social support network was assessed based on the mother’s perception of support received from family, friends, community members, or institutions, and operationalized as a dichotomous variable (no/yes), reflecting the presence of any reported support [42, 43].

The breastfeeding variable comprised two dimensions: breastfeeding (BF) and exclusive breastfeeding (EBF). Both were analyzed as dichotomous variables (no/yes), according to the practices reported by mothers at the time of data collection.

### Statistical analysis

**Fig 1:**
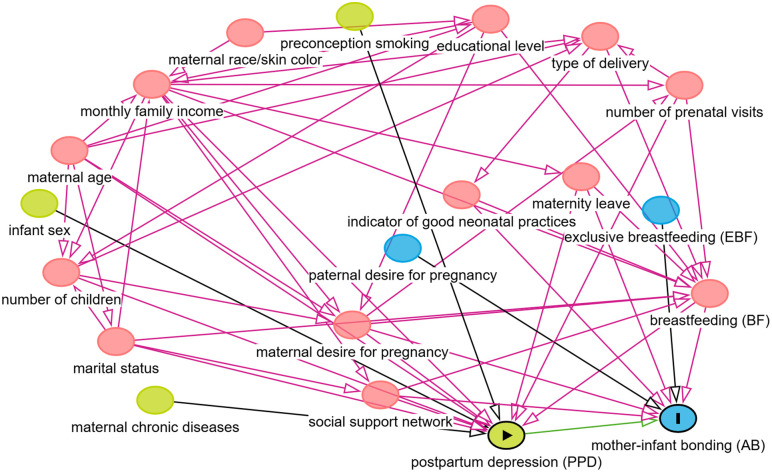
Theoretical-conceptual model of the predictors of the mother-infant emotional bond.

Categorical data were described using absolute and relative frequencies, while numerical data were summarized as means and standard deviations. Statistically significant differences between variables related to PPD and AB were assessed using the Chi-square or Fisher’s exact test for categorical variables and the Wilcoxon test for numerical variables. A linear regression model was employed to analyze variations in AB scores concerning the set of explanatory variables, with the final model selected via the stepwise method. Variable selection for the regression model was guided by systematic literature reviews and theoretical considerations, aiming to include factors recognized as determinants of the mother-infant bond. A Directed Acyclic Graph (DAG) was utilized as a visual tool to organize and clarify hypotheses regarding relationships among study variables [44] (Fig 1).

Statistical analyses were conducted using RStudio (version 2023.03.0 + 386) and IBM SPSS Statistics version 23 for Windows. The choice of software was based on the research team’s expertise and preferences. A significance level of 0.05 (p < 0.05) and 95% confidence intervals were applied for all analyses.

### Ethical issues

The study was approved by the Research Ethics Committee of MEAC-UFC (protocol number 5861734), in accordance with the ethical principles outlined in Resolution 466/2012 of the Brazilian National Health Council (CNS) [45]. All participants provided written informed consent. The ethics committee approval included explicit authorization for telephone follow-up. Informed consent was obtained in person, and participant confidentiality was ensured through numerical identifiers and secure data storage. Data were collected using study-specific forms and recorded in REDCap.

## Results

Tables 1 and 2 present the main characteristics of the participants. The majority resided in the state capital (90%), identified as black or brown (86%), were aged 35 years or older (22%), and had incomplete secondary education (38%). Nearly half reported a family income of up to one minimum wage (49%), and most had more than one child (65%). Regarding family planning, most mothers indicated that neither they (64%) nor the fathers (60%) desired the pregnancy. Approximately 14% reported smoking prior to pregnancy. Most mothers attended six or more prenatal consultations (87%), cesarean deliveries were predominant (68%), and about 26% reported indicators of good neonatal practices (skin-to-skin contact and breastfeeding at birth). The mean duration of BF was 5.4 ± 1.2 months, and EBF lasted an average of 3.7 ± 2.1 months.

**Table 1:** Univariate analysis of AB, PPD symptoms, and sociodemographic, obstetric, and neonatal characteristics at T1 (third month postpartum), 2024.

Characteristics	¹n/ %	^a^AB total – bonding disorder			^b^ABF1- impaired bonding			^c^ABF3- anxiety about the infant		
		compromised n¹/% N=81	preserved n¹/% N=44	*P*²	compromised n¹/% N=84	preserved n¹/% N=41	*P*²	compromised n¹/% N=54	preserved n¹/% N=71	*P*²
**Maternal age**										
≥35	27 (22)	20 (25)	7 (16)	0.254	23 (27)	4 (9.8)	**0.025**	16 (30)	11 (15)	0.057
<35	98 (78)	61 (75)	37 (84)		61 (73)	37 (90)		38 (70)	60 (85)	
**Educational level**										
incomplete	48 (38)	33 (41)	15 (34)	0.465	35 (42)	13 (32)	0.282	19 (35)	29 (41)	0.519
complete	77 (62)	48 (59)	29 (66)		49 (58)	28 (68)		35 (65)	42 (59)	
**Marital status**				0.379			0.513			0.543
Does not live with partner	19 (15)	14 (17)	5 (11)		14 (17)	5 (12)		7 (13)	12 (17)	
Lives with partner	106 (86)	67 (83)	39 (89)		70 (83)	36 (88)		47 (87)	59 (83)	
**Maternal chronic diseases**										
Yes	42 (34)	30 (37)	12 (27)	0.270	32 (38)	10 (24)	0.128	20 (37)	22 (31)	0.478
No	83 (66)	51 (63)	32 (73)		52 (62)	31 (76)		34 (63)	49 (69)	
**Paternal desire for pregnancy**										
No	75 (60)	51 (63)	24 (55)	0.359	54 (64)	21 (51)	0.162	33 (61)	42 (59)	0.825
Yes	50 (40)	30 (37)	20 (45)		30 (36)	20 (49)		21 (39)	29 (41)	
**Maternity leave**										
No	98 (78)	68 (84)	30 (68)	**0.041**	69 (82)	29 (71)	0.146	42 (78)	56 (79)	0.883
Yes	27 (22)	13 (16)	14 (32)		15 (18)	12 (29)		12 (22)	15 (21)	
**Type of delivery**										
Cesarean	86 (69)	56 (69)	30 (68)	0.912	58 (69)	28 (68)	0.932	41 (76)	45 (63)	0.134
Vaginal	39 (31)	25 (31)	14 (32)		26 (31)	13 (32)		13 (24)	26 (37)	
**Indicator of good neonatal practices**										
No	93 (74)	57 (70)	36 (82)	0.161	60 (71)	33 (80)	0.276	42 (78)	51 (72)	0.450
Yes	32 (26)	24 (30)	8 (18)		24 (29)	8 (20)		12 (22)	20 (28)	
^**d**^ **EBF**										
No	41 (34)	34 (44)	7 (16)	**0.002**	34 (43)	7 (18)	**0.006**	21 (42)	20 (29)	0.126
Yes	79 (66)	43 (56)	36 (84)		46 (58)	33 (83)		29 (58)	50 (71)	
**Social support network**				0.097			0.164			0.327
No	10 (8)	9 (11)	1 (2.3)		9 (11)	1 (2.4)		6 (11)	4 (5.6)	
Yes	115(92)	67 (91)	40 (95)		75 (89)	40 (98)		48 (89)	67 (94)	
**Postpartum depression**										
Yes	105 (84)	81 (100)	24 (55)	**<0.001**	82 (98)	23 (56)	**<0.001**	54 (100)	51 (72)	**<0.001**
No	20 (16)	0 (0)	20 (45)		2 (2.4)	18 (44)		0 (0)	20 (28)	

Table 1. Univariate analysis for AB at T1 (third month postpartum).

^a^AB total = affective bonding total.

^b^ABF1 = affective bonding factor 1.

^c^ABF3 = affective bonding factor 3.

^d^EBF = exclusive breastfeeding.

¹n (%); Mean ± Standard Deviation ²2Fisher’s exact test; Wilcoxon rank sum test; chi-square test of independence.

Source- Prepared by the authors.

**Table 2:** Univariate analysis of AB, PPD symptoms, and sociodemographic, obstetric, and neonatal characteristics at T2 (sixth month postpartum), 2024.

Characteristics	¹n/ %	^a^AB total- bonding disorder			^b^ABF1- impaired bonding			^c^ABF3- anxiety about the infant		
		compromised n¹/% N=25	preserved n¹/% N=91	*P*²	compromised n¹/% N=42	preserved n¹/% N=74	*P*²	compromised n¹/% N=4	preserved n¹/% N=112	*P*²
**Maternal age**										
≥35	25 (22)	6 (24)	19 (21)	0.737	11 (26)	14 (19)	0.360	2 (50)	23 (21)	0.203
<35	91 (78)	19 (76)	72 (79)		31 (74)	60 (81)		2 (50)	89 (79)	
**Educational level**										
incomplete	44 (38)	11 (44)	33 (36)	0.480	17 (40)	27 (36)	0.670	0 (0)	44 (39)	0.296
complete	72 (62)	14 (56)	58 (64)		25 (60)	47 (64)		4 (100)	68 (61)	
**Marital status**				0.240			**0.031**			0.516
Does not live with partner	19 (16)	6 (24)	13 (14)		11 (26)	8 (11)		1 (25)	18 (16)	
Lives with partner	97 (84	19 (76)	78 (86)		31 (74)	66 (89)		3 (75)	94 (84)	
**Maternal chronic diseases**										
Yes	41 (35)	9 (36)	32 (35)	0.938	13 (31)	28 (38)	0.456	1 (25)	40 (36)	>0.999
No	75 (65)	16 (64)	59 (65)		29 (69)	46 (62)		3 (75)	72 (64)	
**Paternal desire for pregnancy**										
No	69 (59)	16 (64)	53 (58)	0.603	28 (67)	41 (55)	0.235	4 (100)	65 (58)	0.146
Yes	47 (41)	9 (36)	38 (42)		14 (33)	33 (45)		0 (0)	47 (42)	
**Maternity leave**										
No	90 (78)	18 (72)	72 (79)	0.450	31 (74)	59 (80)	0.462	3 (75)	87 (78)	>0.999
Yes	26 (22)	7 (28)	19 (21)		11 (26)	15 (20)		1 (25)	25 (22)	
**Type of delivery**										
Cesarean	79 (68)	14 (56)	65 (71)	0.143	23 (55)	56 (76)	**0.020**	3 (75)	76 (68)	>0.999
Vaginal	37 (32)	11 (44)	26 (29)		19 (45)	18 (24)		1 (25)	36 (32)	
I**ndicator of good neonatal practices**										
No	86 (74)	17 (68)	69 (76)	0.429	25 (60)	61 (82)	**0.007**	3 (75)	83 (74)	>0.999
Yes	30 (26)	8 (32)	22 (24)		17 (40)	13 (18)		1 (25)	29 (26)	
^**d**^ **EBF**										
No	69 (59)	14 (56)	55 (60)	0.689	27 (64)	42 (57)	0.427	1 (25)	68 (61)	0.302
Yes	47 (41)	11 (44)	36 (40)		15 (36)	32 (43)		3 (75)	44 (39)	
**Social support network**				**0.022**			0.281			0.279
No	9(7.8)	5 (20)	4 (4.4)		5 (12)	4 (5.4)		1 (25)	8 (7.1)	
Yes	107(92)	20 (80)	87 (96)		37 (88)	70 (95)		3 (75)	104 (93)	
**Postpartum depression**										
Yes	18 (16)	10 (40)	8 (8.8)	**<0.001**	12 (29)	6 (8.1)	**0.003**	4 (100)	14 (13)	**<0.001**
No	98 (84)	15 (60)	83(91)		30 (31)	68 (69)		0 (0)	98 (88)	

Table 2. Univariate analysis for AB at T2 (sixth month postpartum).

^a^AB total = affective bonding total.

^b^ABF1 = affective bonding factor 1.

^c^ABF3 = affective bonding factor 3.

^d^EBF = exclusive breastfeeding.

¹n (%); Mean ± Standard Deviation ²2Fisher’s exact test; Wilcoxon rank sum test; chi-square test of independence.

Source- Prepared by the authors.

Variables statistically associated with AB at three months postpartum (T1) included maternity leave (p = 0.041) in the AB total score, EBF in both the AB total score (p = 0.002) and the impaired bonding factor (ABF1) (p = 0.006), and maternal age (p = 0.025) in ABF1. PPD also showed statistically significant associations with the AB total score (p = 0.001), the impaired bonding factor (ABF1) (p = 0.001), and the anxiety about the infant factor (ABF3) (p = 0.001) (Table 1).

At six months postpartum (T2), variables statistically associated with AB included the social support network (p = 0.022) in the AB total score, marital status (p = 0.031), type of delivery (p = 0.020), and indicators of good neonatal practices (p = 0.007) in the impaired bonding factor (ABF1). PPD also showed statistically significant associations with the AB total score (p = 0.001), the impaired bonding factor (ABF1) (p = 0.003), and the anxiety about the infant factor (ABF3) (p = 0.001) (Table 2).

### Multiple linear regression, Stepwise Backward model

The multivariate analysis included the following variables: PPD score, place of residence, maternal race/skin color, maternal age, educational level, maternal chronic diseases, marital status, monthly family income, maternity leave, number of children, type of delivery, maternal and paternal desire for pregnancy, number of prenatal visits, preconception smoking, infant sex, indicator of good neonatal practices, social support network, and EBF.

At three months postpartum (T1), variables statistically associated with AB included maternal chronic diseases (β = 1.84, 95% CI: 0.15–3.53) in the impaired bonding factor (ABF1), and maternal age (β = 0.06, 95% CI: 0.01–0.10) in the anxiety about the infant factor (ABF3). PPD also showed statistically significant associations with the AB total score (β = 1.15, 95% CI: 0.93–1.37), the impaired bonding factor (ABF1) (β = 0.59, 95% CI: 0.46–0.72), and the anxiety about the infant factor (ABF3) (β = 0.28, 95% CI: 0.23–0.33).

At six months postpartum (T2), variables statistically associated with AB included maternal age (β = 0.22, 95% CI: 0.04–0.41), educational level (β = 3.63, 95% CI: 1.17–6.09), paternal desire for pregnancy (β = 2.52, 95% CI: 0.14–4.89), and social support network (β = –8.53, 95% CI: –13.03 to –4.04) in the AB total score, maternal chronic diseases (β = 1.88, 95% CI: 0.33–3.44) and social support network (β = –3.59, 95% CI: –6.26 to –0.93) in the impaired bonding factor (ABF1), and maternal age (β = 0.05, 95% CI: 0.01–0.09) and indicators of good neonatal practices (β = 0.88, 95% CI: 0.15–1.61) in the anxiety about the infant factor (ABF3). PPD also showed statistically significant associations with the AB total score (β = 0.84, 95% CI: 0.61–1.08), the impaired bonding factor (ABF1) (β = 0.40, 95% CI: 0.26–0.54), and the anxiety about the infant factor (ABF3) (β = 0.24, 95% CI: 0.19–0.30).

The direction of the association is indicated by the sign of the regression coefficient (β): positive values reflect higher PBQ scores (worse mother–infant bonding), whereas negative values reflect lower PBQ scores (better bonding). Thus, PPD was associated with worsening bonding across all domains at both T1 and T2, while the social support network was associated with improved bonding in the AB total score and impaired bonding factor (ABF1) at T2 (Table 3).

**Table 3:** Multiple linear regression of AB and predictors at T1 (third month postpartum) and T2 (sixth month postpartum), 2024.

	T1						T2					
	AB total – bonding disorder		ABF1- impaired bonding		ABF3- anxiety about the infant		AB total- bonding disorder		ABF1- impaired bonding		ABF3- anxiety about the infant	
Variáveis	β (IC95%)	p	β (IC95%)	p	β (IC95%)	p	β (IC95%)	p	β (IC95%)	p	β (IC95%)	p
Cesarean	0.48 (−2.33–3.29)	0.736	−0.75 (−2.49–0.98	0.393	−0.16 (−0.81–0.50)	0.641	−1.01 (−3.50–1.48)	0.422	−1.20 (−2.78–0.38)	0.135	0.16 (−0.53–0.84)	0.653
Score Postpartum depression	1.15 (0.93–1.37)	**<0.001**	0.59 (0.46–0.72)	**<0.001**	0.28 (0.23–0.33)	**<0.001**	0.84 (0.61–1.08)	**<0.001**	0.40 (0.26–0.54)	**<0.001**	0.24 (0.19–0.30)	**<0.001**
Maternal Age	–	–	–	–	0.06 (0.01–0.10)	**0.020**	0.22 (0.04–0.41)	**0.019**	–	–	0.05 (0.01–0.09)	**0.015**
Maternal chronic diseases	–	–	1.84 (0.15–3.53)	**0.034**	–	–	–	–	1.88 (0.33–3.44)	**0.018**	–	–
Educational level incomplete	–	–	–	–	–	–	3.63 (1.17–6.09)	**0.004**	–	–	–	–
Paternal desire for pregnancy	–	–	–	–	–	–	2.52 (0.14–4.89)	**0.038**	–	–	–	
Social support network	–	–	–	–	–	–	−8.53 (−13.03 – −4.04)	**<0.001**	−3.59 (−6.26 – −0.93)	**0.009**		
Indicator of good neonatal practices	–	–	–	–	–	–	–	–	–	–	0.88 (0.15–1.61)	**0.019**
R^2^	0.46		0.41		0.51		0.39		0.28		0.44	

Table 3. Multiple linear regression of AB and predictors at T1 (third month postpartum) and T2 (sixth month postpartum).

^a^AB total = affective bonding total

^b^ABF1 = affective bonding factor 1.

^c^ABF3 = affective bonding factor 3.

Source- Prepared by the authors.

## Discussion

The findings underscore the importance of incorporating psychosocial and obstetric factors into postpartum care. Overlooking these elements may have substantial consequences for clinical practice and strategies to strengthen AB. Longitudinal monitoring of AB risk factors, as conducted in this study, offers an effective approach to promoting healthy bonding from early infancy. Maternal age, educational level, social support network, desire for pregnancy, and maternal chronic diseases emerged as key factors requiring clinical attention. These results align with prior research on the multifaceted nature of bonding quality.

PPD emerged as the main predictor of impaired mother–infant bonding. Significant associations (p < 0.001) were observed between PPD and the AB total score, impaired bonding factor (ABF1), and anxiety about the infant factor (ABF3) at both third (T1) and sixth (T2) months postpartum. These robust associations persisted in multivariate models, consistent with findings from Japan [46], China [12], and Brazil [47].

Maternal mental health affected by PPD can impair the quality of daily interactions between mother and infant due to decreased maternal sensitivity and a reduced ability to respond appropriately to the infant’s needs. This may lead to a weakened bond [26,48,49], increasing the risk of behavioral problems and adversely affecting the child’s emotional and cognitive development [50,51], This situation has been further exacerbated by the COVID-19 pandemic [32].

Longitudinal evaluation of PPD and AB during the puerperium was essential to clarify their temporal interplay. PPD prevalence was 84% at T1 and declined to 16% at T2. Total AB compromise occurred in 65% of participants at T1 and 22% at T2; impaired bonding (ABF1) affected 67% and 36% at these time points, respectively. Anxiety about the infant (ABF3) was reported by 43% of women at T1, decreasing to 3% at T2. These findings reveal that PPD exacerbates difficulties in establishing healthy AB, especially at T1, reinforcing the concept of this period as a critical window for bonding development. The gradual improvement in PPD and AB scores suggests that other factors, such as socioeconomic status, obstetric variables, and social support, also play significant roles [19,52,53].

Social support networks are recognized protective factors that alleviate the adverse effects of PPD [19,54]. In this cohort, 92% of breastfeeding women reported receiving support from their social networks, which demonstrated an inverse association with AB across multiple time points and dimensions. This underscores the role of social support in enhancing mother-infant bonding quality. The presence of a social support network appears to facilitate maternal adaptation to puerperal changes, affirming its significance as a protective factor during this period and aligning with prior research [6,55]. Adequate support enables mothers to allocate more time to infant care and self-care, whereas its absence often leads to increased maternal exhaustion [4]. Additionally, mothers not cohabiting with their partners exhibited a higher prevalence of bonding difficulties, potentially heightening vulnerability to stress and depressive symptoms, thereby adversely affecting maternal functioning and infant interaction [6,56].

Sociodemographic factors, including maternal age and education, were linked to poorer AB quality in this study. Similar results were reported by Cavalcante et al. (2017) [47], Doyle et al. (2023) [55]  and Souza (2019) [13]. A systematic review of 15 studies found that seven reported lower AB among older mothers, while six found no significant association. In this study, advanced maternal age emerged as a risk factor for AB, associated with less positive maternal interactions, reduced maternal performance [57], and decreased smiling frequency during mother-infant exchanges [58]. Older mothers may experience greater emotional vulnerability and reduced maternal engagement, possibly due to prior adverse experiences such as childcare challenges or fetal loss. They may also face career interruptions or complications related to late pregnancy. Additionally, this group often reports elevated stress levels [52,59] and heightened expectations regarding their infant’s health and well-being, reflecting increased responsibility and maternal awareness [60,61].

Low maternal education was identified as an additional risk factor for poorer AB quality, compromising adaptation to the puerperium and daily life organization. This association may reflect limited access to health information and essential child-rearing resources [13,14,47], as well as limited maternal knowledge of effective care and stimulation practices that promote child development during emotional interactions [35]. These findings underscore the fundamental need for public policies prioritizing health education, alongside social and psychological support for mothers in vulnerable situations.

The impact of maternal age and education on mother-infant bonding is multifactorial, influenced by various maternal and contextual determinants. In socially vulnerable settings, the numerous demands of motherhood may impair maternal responsiveness, consequently diminishing the emotional availability and sensitivity essential for establishing secure attachment bonds [46].

The regression model identified factors not initially associated with AB or its subscales in the univariate analysis, including increased ABF1 linked to maternal chronic diseases at T1 and T2, and elevated AB total associated with paternal desire for pregnancy being absent at T2. Maternal chronic diseases may induce emotional instability, reduced self-esteem, and diminished maternal self-efficacy by intensifying the demanding care routine for the newborn. The additional burden of managing chronic conditions affects mother-infant bonding due to the necessity of ongoing screening and specialized care beginning in the prenatal period [17,62,63].

Absence of paternal desire for pregnancy has been associated with poorer AB quality [47,61]. This may result from inadequate emotional and financial preparation or trigger ambivalent emotions including love and hate, acceptance and rejection, or guilt, thus increasing maternal burden [15,64]. These findings underscore the importance of partner agreement on family planning [65,66] and active paternal engagement during pregnancy, delivery, and postpartum in promoting optimal AB quality [67,68]. Under such circumstances, social support networks play a pivotal role in safeguarding maternal mental health. Emotional support from family, friends, and healthcare professionals can alleviate psychological distress and enhance maternal autonomy, promoting sensitive, responsive caregiving critical to establishing secure mother-infant bonding [68,69].

A key finding regarding AB involves the maternal anxiety subscale (ABF3) related to infant care, which was linked to the indicator of good birth practices—specifically, skin-to-skin contact and immediate breastfeeding—assessed at six months postpartum. This variable showed a significant association with higher AB scores, indicating a decline in mother-infant bonding quality. Although these neonatal practices are typically associated with improved AB outcomes [6,18,70], it is crucial to acknowledge that during the early postpartum period, physical discomfort from the puerperium and the emotional intensity surrounding childbirth [6] may elevate maternal anxiety. This anxiety may stem from perceived pressure to meet idealized caregiving expectations, feelings of inadequacy [71], the concept of “romanticized” motherhood [4], and challenges in balancing breastfeeding with other social roles [72].

Finally, some factors demonstrated significant associations in univariate analyses but did not retain significance in multivariate models, including maternity leave, breastfeeding, and marital status. This discrepancy reflects the complex interplay among variables influencing AB, where effects may be mediated or moderated by critical factors such as PPD—a key risk factor—and social support networks, which serve as essential protective factors in predicting AB.

Despite this, these variables remain important due to their substantial economic and social implications. Studies by Doyle et al. (2023) [55] and Braga et al. (2021) [73] revealed that the absence of maternity leave adversely affected AB, resulting in reduced time for infant care, breastfeeding, financial support, and increased maternal stress, underscoring the vulnerability of maternal work support, precarious employment conditions, and the lack of guaranteed rights for many women [15,16]. Likewise, EBF was associated with AB quality. Research by Nakano et al. (2020) [74], Doyle et al. (2023) [55] and Nascimento et al. (2021) [75] indicated that negative breastfeeding experiences may induce anxiety, feelings of disappointment, and failure, which can impair maternal responsiveness and bonding capacity [76].

This study has some limitations that warrant consideration, particularly those related to participant follow-up. The sample size may have been insufficient to detect certain significant associations. Losses to follow-up are common in longitudinal research and primarily resulted from changes in address or telephone contact, unavailability, or lack of participant interest. Additionally, potential recall bias should be acknowledged, as participants may have struggled to accurately report their feelings or may have underreported or overestimated symptoms due to fear of judgment, especially during the puerperium—a period marked by substantial change. The variable ‘paternal desire for pregnancy,’ assessed indirectly via maternal report, introduces potential information bias; however, reliance on the mother’s perception was justified given its direct relevance to AB. Furthermore, the use of the EPDS beyond its original validation window—recommended from two weeks postpartum—may reduce sensitivity for early-onset cases. Nevertheless, longitudinal studies by Santos [41] and Figueira [77] have validated its application from the third month postpartum for identifying late-onset PPD.

Future research should incorporate qualitative methods to deepen the understanding of the lived experiences of mothers facing PPD and challenges in AB. These approaches can uncover underlying themes, sociocultural factors, and experiential dimensions that quantitative measures alone may not fully capture. By exploring maternal perceptions and narratives, qualitative research can highlight emotional complexities, social pressures, and contextual barriers influencing maternal bonding. This enriched perspective will enhance data interpretability and practical applicability, supporting the development of culturally sensitive interventions and public policies tailored to the diverse needs of postpartum women. Ultimately, integrating qualitative insights will advance comprehensive knowledge of the AB-PPD relationship, promoting improved maternal and infant health outcomes.

## Conclusion

This study aimed to identify factors associated with mother-infant bonding (AB) at three (T1) and six (T2) months postpartum in a low-risk obstetric and neonatal cohort. It revealed a significant association between PPD and other risk factors associated with compromised AB. These findings underscore the need for a multifactorial approach during the postpartum period—a critical window for affective bond consolidation—and emphasize prioritizing PPD as a universal intervention target, even in populations traditionally considered low-risk. Longitudinally, at three months postpartum (T1), PPD emerged as the strongest predictor of compromised AB, alongside significant influences from maternal age and chronic maternal diseases. By six months postpartum (T2), in addition to PPD, adverse socioeconomic factors—such as incomplete education and paternal unwanted pregnancy—were linked to greater challenges in strengthening AB. Conversely, a robust social support network emerged as a key protective factor.

These findings have significant implications for clinical practice and public policy, underscoring the necessity of comprehensive interventions that address maternal mental health, social context, and lived experiences during childbirth and the puerperium. Furthermore, the observed improvement in PPD and AB indicators at T2 emphasizes the critical role of extended monitoring throughout the puerperal period. Such follow-up is vital not only for the early identification of bonding difficulties but also for the timely implementation of intervention strategies aimed at fostering maternal sensitivity and enhancing the quality of daily mother-infant interactions. These efforts ultimately strengthen AB and mitigate potential disruptions in this essential relationship.

A comprehensive understanding of the interplay among biological, social, and psychological factors is essential for holistic maternal and child healthcare. This perspective underscores the need for further research to elucidate how these dimensions interact across diverse contexts and populations, as well as to develop integrated intervention strategies that combine psychosocial support, systematic screening, and early management of PPD with continuous assessment of mother-infant bonding.

## Supporting information

S1 File Set of data and metadata (CSV) analyzed in the study.
(CSV)

S2 File Report from the ethics committee indicating approval of the research.
(PDF)
