# Supplementary material for: Postpartum depression as the main predictor of impaired mother- infant bonding: A prospective study in a low-risk obstetric and neonatal cohort
Source: PLoS One. 2026 May 29;21(5):e0347856. doi: 10.1371/journal.pone.0347856 (PMC13221017; doi:10.1371/journal.pone.0347856)
Supplement: S2 File — Report from the ethics committee indicating approval of the research. (PDF) [file pone.0347856.s002.pdf]

CONSUBSTANTIATED OPINION OF THE CEP

RESEARCH PROJECT DATA

Search Title: SYMPTOMS OF ANXIETY AND DEPRESSION, MOTHER-BABY AFFECTIONATE BOND, BREASTFEEDING SELF-EFFICACY: A COHORT STUDY IN THE FIRST SIX MONTHS POSTPARTUM

Researcher: MARIELLE RIBEIRO FEITOSA

Thematic Area:

Version: 1

CAAE: 66182722.4.0000.5050

Proposing Institution: Maternity School Assis Chateaubriand / MEAC/ UFC

Main Sponsor: Own Financing

OPINION DATA

Opinion Number: 5,861,734

Project Presentation: The information listed in the fields “Project Presentation”, “Research Objectives” and “Risk and Benefit Assessment” were taken from the Basic Research Information file (PB_INFORMAÇÕES_BÁSICAS_DO_PROJETO_2055810.pdf, submitted on 12/19/2022.

This is a project entitled "ANXIETY AND DEPRESSION SYMPTOMS, MOTHER-BABY AFFECTIVE BOND, BREASTFEEDING SELF-EFFICACY: A COHORT STUDY IN THE FIRST SIX MONTHS POSTPARTUM".

It is a prospective cohort study to be carried out in a public maternity hospital in the State of Ceará.
The sequential sampling model was chosen because it allows recruiting all individuals from a population accessible to assistance at the institution and who meet the eligibility criteria over a specific time interval.

Breastfeeding mothers with their respective full-term newborns, admitted to the institution with the following criteria, will participate: age 18 years, 0 to 30 days postpartum, exclusively breastfed, with no obstetric or neonatal complications related to birth.

Data collection consists of retrieving information from the mothers' medical records, filling out a form during their stay at the institution, and inviting them to participate in other telephone contacts to answer some questions about how they are continuing with breastfeeding in the first, third and sixth months of their child's life.

Research Objective:

Primary Objective:

• To assess symptoms of anxiety and depression, the mother-baby emotional bond and breastfeeding self-efficacy in the first six months postpartum and their associations with the duration of EBF.

Secondary Objective:

• Assess the prevalence of maternal anxiety and depression symptoms in the first six months postpartum, according to selected variables;

• To assess the mother-baby emotional bond in the first six months postpartum, according to selected variables; • To assess the level of self-efficacy for breastfeeding in the first six months postpartum, according to selected variables;

• Verify the correlation between the independent variables: maternal anxiety and depression symptoms, the mother-baby emotional bond and breastfeeding self-efficacy;

• To assess the mediating role of the mother-baby emotional bond in the relationship between maternal anxiety and depression symptoms and the duration of EBF.

Risk and Benefit Assessment:

Risks:

Responding to this survey form may expose you to risks such as tiredness and discomfort from hospitalization and from remembering situations with your newborn that may trigger your feelings. To reduce the risk, we will try to collect this information when you are feeling comfortable, in a calm place and, if necessary, we will ask for the support of the psychologist and also of a family member with whom you feel safe and calm.

Address: Cel Nunes de Melo Street, s/n
Neighborhood: Rodolfo Theophilus **ZIP Code:** 60.430-270
State: CE **Municipality:** FORTALEZA
Telephone: (85)3366-8569 **Fax:** (85)3366-8528 **Email:** cepmeac@gmail.com

Benefits:

The information obtained may be useful for us to verify the potential of the care received here at the institution regarding breastfeeding, the influence on breastfeeding and the guarantee of positive effects for your baby.
baby.

This clinical monitoring may allow early identification of possible practices/care
received by you or not that can be improved, bringing greater benefits such as support in the exclusive breastfeeding of your children to the next mothers and babies who came to be admitted to this
maternity.

Comments and Considerations on the Research:

Academic study is part of the Teaching Department and the POSTGRADUATE PROGRAM IN APPLIED RESEARCH IN CHILDREN'S AND WOMEN'S HEALTH to obtain the title of Doctorate.

Carried out at the Assis Chateaubriand Maternity School (MEAC) and the Brazilian Hospital Services Company (EBSERH) of the Federal University of Ceará, in Fortaleza, with funding from the researcher.

Data collection will take place during the year 2023 with the completion of the thesis in 2025.

Considerations on Mandatory Presentation Terms: All mandatory terms
have been presented appropriately.
See field “Conclusions or Pending Issues and List of Inadequacies”

Recommendations:

Project without recommendations
See field “Conclusions or Pending Issues and List of Inadequacies”

Conclusions or Pending Issues and List of Inadequacies:

APPROVED Project

Final Considerations at the discretion of the CEP:

It is important to note that it is up to the responsible researcher to submit the partial and final reports of the

Address: Cel Nunes de Melo Street, s/n
Neighborhood: Rodolfo Theophilus **ZIP Code:** 60.430-270
State: CE **Municipality:** FORTALEZA
Telephone: (85)3366-8569 **Fax:** (85)3366-8528 **Email:** cepmeac@gmail.com

research, through the Brazil Platform, via “report” type notification so that they can be duly assessed by the CEP, according to CNS Operational Standard No. 001/13, item XI.2.d.

This opinion was prepared based on the documents listed below:

Document Type	File	Post	Author	Situation
Declaration of Institution and Infrastructure	Letterofannouncementpdf.pdf	12/19/2022 08:37:29	Maria Irilandia Keys	Accepted
ROJETO_2055810.pdf Basic Project Information	PB_INFORMAÇÕES_BÁSICAS_DO_P	12/19/2022 08:04:16		Accepted
Termosciencepdf.pdf		12/19/2022 08:03:53	MARIELLE RIBEIRO MADE	Accepted
Timeline	ProjectSchedule.pdf	12/16/2022 17:55:59	MARIELLE RIBEIRO MADE	Accepted
Declaration of agreement	DECLARACAODECONCORDANCIA.pdf 16/12/2022 17:52:50	16/12/2022	MARIELLE RIBEIRO MADE	Accepted
Detailed Design / Brochure Researcher	EbserhResearch projectMarielleRibeiro Feitosa.pdf	17:51:27	MARIELLE RIBEIRO MADE	Accepted
TCLE / Terms of Assent / Justification of Absence	TCLEplatform.pdf	12/16/2022 5:48:57 PM	MARIELLE RIBEIRO MADE	Accepted
Title Page	EbserhSignedFacebook.pdf	12/16/2022 5:47:05 PM	MARIELLE RIBEIRO MADE	Accepted

Opinion Status:

Approved

Needs CONEP's Appreciation:

No

FORTALEZA, January 23, 2023

Signed by:
Maria Sidneuma Melo Ventura
(Coordinator)

PARECER CONSUBSTANCIADO DO CEP

DADOS DO PROJETO DE PESQUISA

Título da Pesquisa: SINTOMAS DE ANSIEDADE E DEPRESSÃO, VÍNCULO AFETIVO MÃE ¿BEBÊ, AUTOEFICÁCIA NA AMAMENTAÇÃO: UM ESTUDO DE COORTE NOS PRIMEIROS SEIS MESES PÓS-PARTO

Pesquisador: MARIELLE RIBEIRO FEITOSA

Área Temática:

Versão: 1

CAAE: 66182722.4.0000.5050

Instituição Proponente: Maternidade Escola Assis Chateaubriand / MEAC/ UFC

Patrocinador Principal: Financiamento Próprio

DADOS DO PARECER

Número do Parecer: 5.861.734

Apresentação do Projeto:

As informações elencadas nos campos “Apresentação do Projeto”, “Objetivos da Pesquisa” e “Avaliação dos Riscos e Benefícios” foram retiradas do arquivo Informações Básicas da Pesquisa (PB_INFORMAÇÕES_BÁSICAS_DO_PROJETO_2055810.pdf, submetido 19/12/2022.

Trata-se de um projeto intitulado "SINTOMAS DE ANSIEDADE E DEPRESSÃO, VÍNCULO AFETIVO MÃE–BEBÊ, AUTOEFICÁCIA NA AMAMENTAÇÃO: UM ESTUDO DE COORTE NOS PRIMEIROS SEIS MESES PÓS-PARTO".

É uma Pesquisa de Coorte prospectiva a ser realizada em uma maternidade pública no Estado do Ceará. Foi escolhido o modelo de amostragem em sequência, pois permite recrutar todos os indivíduos de uma população acessível a assistência na instituição e que atendam aos critérios de elegibilidade ao longo de um intervalo de tempo específico.

Participarão as lactantes com seus respectivos recém-nascidos a termo, admitidos na instituição com os seguintes critérios: idade 18 anos, estejam no pós-parto de 0 a 30 dias, em amamentação exclusiva, com ausência de complicações obstétricas ou neonatais relacionadas ao nascimento.

A coleta de dados consiste no resgate de informações nos prontuários das respectivas mães, o preenchimento de um formulário durante a internação na instituição e serão convidadas a participarem de outros contatos por telefone para responder algumas perguntas sobre como estão seguindo com o aleitamento no primeiro, terceiro e sexto mês de vida do seu filho.

Objetivo da Pesquisa:

Objetivo Primário:

• Avaliar os sintomas de ansiedade e depressão, o vínculo afetivo mãe-bebê e a autoeficácia em amamentar nos primeiros seis meses pós-parto e suas associações com o tempo de duração do AME.

Objetivo Secundário:

• Avaliar a prevalência de sintomas de ansiedade e depressão maternos nos primeiros seis meses pós-parto, de acordo com variáveis selecionadas;
• Avaliar o vínculo afetivo mãe-bebê nos primeiros seis meses pós-parto, de acordo com variáveis selecionadas;
• Avaliar o nível de autoeficácia para amamentar nos primeiros seis meses pós-parto, de acordo com variáveis selecionadas;
• Verificar a correlação entre as variáveis independentes: sintomas de ansiedade e depressão maternos, o vínculo afetivo mãe-bebê e autoeficácia em amamentar;
• Avaliar o papel mediador do vínculo afetivo mãe-bebê na relação entre os sintomas de ansiedade e depressão maternos com o tempo de duração do AME.

Avaliação dos Riscos e Benefícios:

Riscos:

Responder ao formulário dessa pesquisa poderá expor você a riscos como cansaço e desconforto da internação e por relembrar situações do seu recém-nascido que podem mobilizar seus sentimentos. Para reduzir o risco tentaremos coletar essas informações quando você estiver se sentindo à vontade, em local calmo e se necessário pedimos o apoio da psicóloga e ainda de um familiar que você se sinta segura e tranquila.

Benefícios:

As informações obtidas poderão ser úteis para verificarmos o potencial dos cuidados recebidos aqui na instituição sobre o aleitamento, a influência no aleitamento e a garantia dos efeitos positivos para o seu bebê.

Este acompanhamento clínico poderá permitir a identificação precoce de possíveis práticas/ cuidados recebidos por vocês ou não que possam ser melhorados trazendo maiores benefícios como o apoio no aleitamento exclusivo dos seus filhos as próximas mães e bebês que vieram a ser internados nesta maternidade.

Comentários e Considerações sobre a Pesquisa:

Estudo de caráter acadêmico faz parte do Departamento de Ensino e do Programa de PÓS-GRADUAÇÃO EM PESQUISA APLICADA SAÚDE DA CRIANÇA E DA MULHER para obtenção do título de Doutorado. Realizado na Maternidade Escola Assis Chateaubriand (MEAC) e Empresa Brasileira de Serviços Hospitalares (EBSERH) da Universidade Federal do Ceará, em Fortaleza, com financiamento do pesquisador.

A coleta de dados ocorrerá durante o ano de 2023 com a finalização da tese em 2025.

Considerações sobre os Termos de apresentação obrigatória:

Todos os termos obrigatórios foram apresentados adequadamente.

Vide campo “Conclusões ou Pendências e Lista de Inadequações

Recomendações:

Projeto sem recomendações

Vide campo “Conclusões ou Pendências e Lista de Inadequações

Conclusões ou Pendências e Lista de Inadequações:

Projeto APROVADO

Considerações Finais a critério do CEP:

Ressalta-se que cabe ao pesquisador responsável encaminhar os relatórios parciais e final da

pesquisa, por meio da Plataforma Brasil, via notificação do tipo “relatório” para que sejam devidamente apreciadas no CEP, conforme Norma Operacional CNS n° 001/13, item XI.2.d.

Este parecer foi elaborado baseado nos documentos abaixo relacionados:

Tipo Documento	Arquivo	Postagem	Autor	Situação
Declaração de Instituição e Infraestrutura	Cartadeanuenciapdf.pdf	19/12/2022 08:37:29	Maria Irilândia Chaves	Aceito
Informações Básicas do Projeto	PB_INFORMAÇÕES_BÁSICAS_DO_PROJETO_2055810.pdf	19/12/2022 08:04:16		Aceito
Outros	Termodecienciapdf.pdf	19/12/2022 08:03:53	MARIELLE RIBEIRO FEITOSA	Aceito
Cronograma	ProjetoCronograma.pdf	16/12/2022 17:55:59	MARIELLE RIBEIRO FEITOSA	Aceito
Declaração de concordância	DECLARACAODECONCORDANCIA.pdf	16/12/2022 17:52:50	MARIELLE RIBEIRO FEITOSA	Aceito
Projeto Detalhado / Brochura Investigador	EbserhProjetodepesquisaMarielleRibeiroFeitosa.pdf	16/12/2022 17:51:27	MARIELLE RIBEIRO FEITOSA	Aceito
TCLE / Termos de Assentimento / Justificativa de Ausência	TCLEplataforma.pdf	16/12/2022 17:48:57	MARIELLE RIBEIRO FEITOSA	Aceito
Folha de Rosto	EbserhFolhadeRostoassinada.pdf	16/12/2022 17:47:05	MARIELLE RIBEIRO FEITOSA	Aceito

Situação do Parecer:
Aprovado

Necessita Apreciação da CONEP:
Não

FORTALEZA, 23 de Janeiro de 2023

Assinado por:
Maria Sidneuma Melo Ventura
(Coordenador(a))

Endereço: Rua Cel Nunes de Melo, s/n
Bairro: Rodolfo Teófilo **CEP:** 60.430-270
UF: CE **Município:** FORTALEZA
Telefone: (85)3366-8569 **Fax:** (85)3366-8528 **E-mail:** cepmeac@gmail.com
